# Immunogenic *Streptococcus equi* cell surface proteins identified by ORFeome phage display

**DOI:** 10.1128/msphere.00626-25

**Published:** 2025-11-25

**Authors:** Joshua Wan, Evan Weldon, Gabriella Ganser, Ellen Ruth A. Morris, Emma V. Hughes, Angela I. Bordin, Philip Alexander Heine, Michael Hust, Noah D. Cohen, Jason J. Gill, Mei Liu

**Affiliations:** 1 Center for Phage Technology, Texas A&M AgriLife Research and Texas A&M University 14736, College Station, Texas, USA; 2 Department of Biochemistry and Biophysics, Texas A&M University 124584, College Station, Texas, USA; 3 Texas A&M Veterinary Medical Diagnostic Laboratory 117328 , College Station, Texas, USA; 4 Equine Infectious Disease Laboratory, Department of Large Animal Clinical Sciences, College of Veterinary Medicine & Biomedical Sciences, Texas A&M University 341072, College Station, Texas, USA; 5 Department of Medical Biotechnology, Institute for Biochemistry, Biotechnology and Bioinformatics, Technische Universität Braunschweig, Braunschweig, Germany; 6 Department of Animal Science, Texas A&M University 199048, College Station, Texas, USA; The University of Texas Medical Branch at Galveston, Galveston, Texas, USA

**Keywords:** *Streptococcus equi*, equine strangles, ORFeome phage display, surface protein

## Abstract

Equine strangles caused by *Streptococcus equi* subspecies *equi* (*S. equi*) remains a significant cause of morbidity and mortality in horses, and there is a need for improved diagnostic and vaccination strategies for addressing this pathogen. ORFeome phage display is a platform that allows for rapid screening for potential antigenic epitopes by construction of phage-displayed peptide libraries. In this study, an *S. equi* ORFeome library was used to screen serum from a panel of 17 horses with known exposure to *S. equi* to identify antigenic bacterial proteins. From this screen, three major *S. equi* proteins were identified: a novel proline-rich repeat domain protein, a serine peptidase, and the M-like protein SeM. These three proteins are predicted to be expressed on the surface of the bacterial cell by the presence of N- and C-terminal signals. The proline-rich repeat protein and serine peptidase were confirmed to be immunogenic by enzyme-linked immunoassay (ELISA) using the recombinant full-length proteins against sera from horses with strangles, horses infected with the related pathogen *S. equi* subsp. *zooepidemicus*, and healthy control horses. Due to the native IgG binding activity of SeM, ELISA against the full-length protein was not conducted, but the specificity of the antibody response against the recovered ORFeome clones was confirmed and an antigenic region identified. Both the proline-rich repeat protein and serine peptidase were found to be highly conserved in global *S. equi* genomes, indicating these proteins may be useful as vaccine candidates against *S. equi* or as diagnostic markers to specifically identify *S. equi* infections in horses.

**IMPORTANCE:**

This work utilized an ORFeome phage display platform to systematically identify antigenic epitopes produced by *Streptococcus equi* subspecies equi (*S. equi*), an important equine pathogen and the causative agent of horses strangles. Three major *S. equi* surface proteins were identified: a novel proline-rich repeat domain protein, a serine peptidase, and the M-like protein SeM. The proline-rich repeat protein and serine peptidase were confirmed to be immunogenic in horses with strangles, and their sequences were shown to be conserved in global *S. equi* genomes, in contrast to their diversity in *S. equi* subsp. zooepidemicus. With the well-characterized *S. equi* immunogenic protein SeM, this paper identified an immunogenic region outside of the reported critical IgG-binding region. This work provides novel insights to the understanding of the *S. equi* immunogenic proteins and provides peptide regions that could serve as vaccine candidates against *S. equi* or as diagnostic markers to specifically identify *S. equi* infections.

## INTRODUCTION

Strangles, an acute upper respiratory disease of horses, is caused by *Streptococcus equi* subspecies *equi* (*S. equi*), and is considered the most common equine infectious respiratory disease (1, 2). The infection is clinically characterized by rapid onset of pyrexia, lethargy, and pharyngitis, which leads to difficulty eating and drinking, and abscess formation in the submandibular and retropharyngeal lymph nodes (3). The disease can lead to severe inflammation and abscess formation that can potentially cause respiratory tract obstruction and asphyxiation, necessitating tracheotomy (2). The mortality rate due to severe complications can reach nearly 10%, with young (age ≤5 years) horses being particularly vulnerable (4). The disease is characterized as highly contagious and is transmitted primarily through direct contact with infectious sputum and fluids, contaminated water troughs and feeders, or other fomites or through uptake of bacteria shed from healthy carrier horses harboring *S. equi* (2, 5, 6).

Outbreaks of strangles are globally common and economically consequential (3). Current control measures for this disease remain challenging. Treatment with antibiotics has limitations and is not generally recommended in the acute phase of disease (2, 7). Diagnostic testing and vaccination measures are, therefore, crucial for preventing the establishment of strangles and for controlling its spread. Of particular significance is the necessity of diagnostic tests that can differentiate between *S. equi* and its closely related evolutionary ancestor, *Streptococcus equi* subspecies *zooepidemicus* (abbreviated as *S. zooepidemicus*), which is considered a commensal of the upper airway of horses but can cause upper and lower respiratory tract disease either as an opportunistic infection or primary pathogen (8). Multiplex PCR and serological diagnostic tests employing immunogenic protein biomarkers and their associated genes unique to *S. equi* have been developed to offer more rapid and specific performance than culture-based methods (6, 9–11). The M protein (SeM) of *S. equi*, also termed fibrinogen-binding protein (FgBP), is a cell wall-associated critical virulence agent that functions to impede phagocytosis and facilitate adherence to host cells in infected animals (12–14). SeM and its associated gene are of particular interest in PCR and serological tests for *S. equi* due to its antigenic ubiquity (15, 16). Notably, significant variations in the M-like protein of *S. equi* have been observed, particularly within the N-terminal region, among different *S. equi* isolates, as evidenced by epidemiological studies (17–19), and truncated versions of M-protein have been identified in *S. equi* isolated from persistently infected carriers (20). To date, 269 alleles have been described for the SeM protein, and variants continue to emerge in outbreaks and in individual horses, suggesting this variation might contribute to evasion of immune responses (18, 21). Another fibrinogen-binding M-like protein (SzPSe) of *S. equi* is distantly related to SeM and is strongly opsonogenic for *S. zooepidemicus,* but not for *S. equi* (16). Enzyme-linked immunoassay (ELISA)-based tests for detecting antibody responses to *S. equi* antigens (including SeM and SzPSe) have been developed and are capable of detecting recent infections or exposure to *S. equi* but with varying degrees of specificity (2, 22–25). In comparison to the poorer specificity reported for ELISA based on whole SeM protein, ELISA based on N-terminal recombinant protein fragments of SEQ2190 (antigen A) and SeM (antigen C) of *S. equi* strain 4047 exhibited enhanced sensitivity and specificity for identifying horses exposed to *S. equi* (26). Current vaccines against strangles utilize either purified SeM antigens (e.g., StrepvaxII by Boehringer Ingelheim), mixed *S. equi* proteins (e.g., Strangvac by Intervacc and Equilis Strep E by Intervet), or live attenuated versions of *S. equi* (e.g., Pinnacle IN by Zoetis). However, Strangvac is not available in the United States, and there is room for improvement pertaining to the efficacy and safety of the other vaccines (2, 22, 27–29).

In an effort to identify novel antigens to improve the diagnosis of and vaccine targets for *S. equi*, the present study describes the use of ORFeome phage display as a systematic approach to identify strangles-specific immunogenic biomarker proteins of *S. equi*. The ORFeome phage display pipeline has been utilized extensively to identify novel antigens of interest (30–32). The creation of an ORFeome phage display that encompasses the complete *S. equi* genome entailed the fusion of *S. equi* random gene fragments with the coat protein III gene (gIII) of M13, followed by packaging into the filamentous M13 phage, a process enabling the expression of the oligopeptides and protein fragments encoded in the *S. equi* genome on the phage surface (30, 33). After this, a panning process was employed to select the oligopeptides and protein fragments targeted by the antibodies present in the serum of horses infected with *S. equi*. Subsequent sequencing of the selected phages provided genomic information, which was then used to reconstruct the epitopes of immunogenic proteins. Using this approach, three cell surface proteins were identified as major immunogenic *S. equi* proteins. The primary protein identified was a novel protein carrying two proline-rich sequence repeat domains, while the other two proteins were the *S. pyogenes* serine protease homolog and the well-characterized SeM protein. The proline-rich repeat domain surface protein identified in this study has the potential to be developed as both a diagnostic marker and a vaccine candidate for strangles caused by *S. equi* due to its high degree of sequence conservation in *S. equi,* but not in *S. zooepidemicus*, and its significant interaction with the strangles-positive sera.

## MATERIALS AND METHODS

### *S. equi* display library construction and packaging

An *S. equi* ORFeome phage display library was constructed following the detailed protocols previously described (33). Briefly, genomic DNA of *S. equi* strain 19-033 from the Equine Infectious Disease Laboratory at Texas A&M University was extracted and fragmented via sonication into a library of short DNA fragments (~100 to 500 bp). The DNA fragments were end-repaired using the Fast DNA End Repair Kit (Thermo Scientific) and ligated into the pHORF3 phagemid vector (34), which was digested with the blunt end digestion enzyme *Pme*I and dephosphorylated. The ligation mixture was purified and transformed into electrocompetent *E. coli* SS320 (Biosearch Technologies), and the library insert rate in the obtained transformants was determined by colony PCR. All transformants were pooled together, and the DNA of the constructed ORFeome libraries (pHORF3 vector carrying the *S. equi* DNA inserts) was extracted and sequenced using NovaSeq Illumina sequencing. The obtained reads (2 × 150 bp paired-end, a mixture of the ORF insert reads and the pHORF3 vector reads) were mapped onto the *S. equi* strain SEE 19-033 genome (NZ_JAHRJJ010000001) using Bowtie2 (35), and the resulting BAM file was used to plot the coverage depth of the reads against the *S. equi* genome by WGSCoveragePlotter (Lindenbaum, P. (2015). *JVarkit: java-based utilities for Bioinformatics*. https://github.com/lindenb/jvarkit). These pooled transformants carrying the fusion of *S. equi* DNA to the coat protein III gene of M13 were used to package the *S. equi* DNA fragments into M13 using Hyperphage (M13KO7 ΔpIII) (Progen, Heidelberg, Germany). The packaged phage particles were concentrated by PEG/NaCl precipitation and resuspension. The resuspended phage library was titered and verified for their insert rate via colony PCR, before being used for panning against equine serum samples.

### Panning and screening-ELISA to identify immunogenic proteins

Serum samples were provided by a repository maintained in the Equine Infectious Disease Laboratory at Texas A&M University. Detailed serum information is listed in Data S3. Panning procedures using the constructed ORFeome library and the horse serum samples were performed as described (33) with minor modifications to the incubation procedures. Preincubation of the serum sample and phage library was performed in a 96-well plate (ThermoFisher MaxiSorp) to reduce the nonspecific binding during panning. For serum preincubation to deplete the phage-binding antibodies, 300 µL of Hyperphage (~ 4 × 10^10^ cfu) was immobilized on two wells of the plate and after blocking and washing steps was used to sequentially incubate with 300 µL diluted serum (1:500 dilution in blocking buffer). For phage library preincubation, the phage library (300 µL of 10^10-11^ cfu/mL titer) was first incubated in one well coated with panning block buffer and then was transferred sequentially to wells coated with a control serum sample to deplete the binding to unrelated serum proteins and nonspecific horse antibodies and coated with anti-horse-IgG to deplete the binding to the anchoring antibody used in panning. The duration for all incubation procedures was for 1 h. For panning, 100 µLof the diluted rabbit anti-horse-IgG (Invitrogen, 1:10,000 dilution) was immobilized onto two wells of the plate. The preincubated serum was transferred to these two wells (150 µL per well) for 1 h and after washing was incubated for 1.5 h with the pre-incubated phage library (150 µL to each well), supplemented with 1 × 10^10^ cfu/mL Hyperphage (for soluble competition). After washing, the phage particles bound to these two panning wells were eluted with trypsin (10 µg/mL, 200 µL per well) and were pooled together to infect a logarithmically growing *E. coli* XL1-Blue MRF’ culture to package into a new phage library using Hyperphage to support the second round of panning. After the second round of panning, serial dilutions of the eluted phage populations were used to infect logarithmically growing *E. coli* XL1-Blue MRF’ culture to obtain isolated colonies. Colonies (92 in total) were randomly picked and cultured in a 96-deep-well plate with the help of Hyperphage to generate monoclonal phage population for screening ELISA.

For the screening ELISA, an ELISA plate (ThermoFisher MaxiSorp) was coated with mouse anti-pVIII monoclonal antibody (Invitrogen, 1:10,000 dilution). After blocking and washing, 50 µL of monoclonal phage population was added together with 50 µL of blocking buffer. After 2 h incubation and then washing, 100 µL horse serum (1:500 dilution in blocking buffer) supplemented with 1 × 10^10^ cfu/mL Hyperphage (for soluble competition) was added to the wells and incubated for 2 h. After washing the plate, rabbit anti-horse-IgG-HRP detection antibody (Sigma, 1:10,000 diluted) was added to the wells and incubated for 1 h. The plate was then washed, and 100 µL/well of TMB (Sigma) ELISA developing solution was added to the wells. The plate was incubated for 30 min before the reaction was stopped by adding 1 N H_2_SO_4_ (100 µL/well). The ELISA signal was detected with a plate reader at 450 nm using 620 nm as a reference wavelength. Various negative controls (controls for serum, detection antibodies, and phage production) were included as described in the detailed protocol (33). Clones that exhibited a significant ELISA signal compared to the negative control were cultured to extract plasmid DNA. The ORF insertion sequence in each extracted plasmid DNA sample was determined via Sanger sequencing. The obtained ORF DNA sequences were translated into protein sequences and were aligned with the *S. equi* strain SEE_19-033 sequence (GenBank accession number JAHRJJ000000000) via BLASTp, to identify the immunogenic proteins in the genome.

### Sequence analysis of the protein homologs in *S. equi* and *S. zooepidemicus* genomes

The immunogenic proteins identified by panning were used to search for their homologs in a collection of *S. equi* and *S. zooepidemicus* genomes. The collection of *S. equi* and *S. zooepidemicus* genomes used for this study is from Bioproject PRJNA736470, plus additional genomes from the public database, and the detailed information is listed in Data S1 and S2, respectively. For the genomes deposited under Bioproject PRJNA736470, assembly contig sequences were retrieved from the NCBI and were made into concatenated BLAST databases using the Make BLAST database tool (Galaxy Version 2.14.1 + galaxy2) on the Galaxy platform (Usegalaxy.eu). The protein sequence of the identified immunogenic surface proteins (MCD3496749.1, MCD3496188.1, and MCD3495510.1) was used to Blast the translated nucleotide database using the NCBI tBLASTn tool (Galaxy Version 2.14.1 + galaxy2), to identify the contigs that contain the target protein homologs. After de-duplicating the hit contig accession numbers, all protein sequences from these identified contigs were retrieved using the NCBI Batch Entrez tool and were made into a BLAST database, to which the immunogenic proteins were blasted against. For the rest of the genomes that are not from Bioproject PRJNA736470 (Data S1 and S2), the total protein FASTA sequences from each genome/assembly were manually downloaded from the NCBI, against which the immunogenic protein to be analyzed (whole protein sequence or the partial sequences containing the immunogenic regions) was BLASTed to identify the homolog in each genome. The identified protein homolog accession numbers from all *S. equi* or *S. zooepidemicus* genomes were deduplicated, and protein sequences were retrieved via NCBI’s Batch Entrez. Protein sequence alignment was done using Clustal Omega Multiple Sequence Alignment (MSA) (36) with default settings. A protein phylogenetic tree was generated using Molecular Evolution Genetics Analysis, MEGA (version 11.0.13) after MUSCLE alignment (37). SignalP-6.0 (https://services.healthtech.dtu.dk/services/SignalP-6.0/) was used to predict the signal peptide sequences.

### Recombinant protein expression and reactivity to equine sera testing

For the two non-SeM surface proteins (accession numbers MCD3496749 and MCD3496188), gene sequences encoding the protein excluding the N-terminal signal peptide and the C-terminal LPXTG cell surface anchor sequences were cloned into pET-30a (+) vector and expressed in *E. coli* Top10 by Genscript. The 6 His-tag was introduced into the amino acid sequence of each protein at the C-terminal to purify the protein by affinity chromatography to an apparent homogeneity (according to polyacrylamide gel electrophoresis). Each purified protein was tested for its binding to horse sera following a similar protocol as described in the screening ELISA above. Briefly, the protein was coated onto the ELISA plate (1 µg per well) at 4°C overnight. After washing and blocking the coated plate, the binding toward equine sera was tested by adding serially diluted (either 2-fold or 10-fold dilution) sera to the protein-coated wells. Rabbit anti-horse-IgG-HRP detection antibody (Sigma, 1:10,000 diluted) was used as the detection antibody with the TMB (Sigma) as the developing solution. All horse sera IgG were quantified using a horse IgG ELISA kit (AFG Bioscience). EC50 values of the ELISA signal vs serum dilutions (serum dilution resulting in a half-maximum signal) were calculated using GraphPad Prism v 10.4.0 (GraphPad Software) under Agonist vs response model (variable slope and 4-point parameters). Groups of EC50 values per serum group were compared by pairwise Kruskal-Wallis tests across control, *S. equi,* and *S. zooepidemicus* groups with Dunn’s correction for multiple comparisons and a *P* value cutoff of 0.05. Statistical analysis was conducted in GraphPad Prism v 10.4.0 (GraphPad Software).

### Validation of the interaction of SeM ORFs with different IgGs by ELISA

The individual SeM ORF clones identified after panning and screening ELISA were further tested for their binding to different IgGs following a protocol similar to the screening ELISA. The produced monoclonal phages displaying each ORF (50 µL phage mixed with 50 µLblocking buffer) were immobilized on an ELISA plate via anchoring mouse anti-pVIII antibody (Invitrogen, 1:10,000 dilution), with negative control wells containing Hyperphage (1 × 10^9^ per well) in 50 µL of the supernatant of the *E. coli* XL1-Blue MRF’ overnight culture mixed with 50 µL blocking buffer. In parallel, Hyperphage (1 × 10^9^ per well) was immobilized on a separate ELISA plate via mouse anti-pVIII antibody, and the wells were used to deplete the nonspecific phage binders in IgG samples to be tested. Horse sera and commercial IgG samples were standardized for their IgG levels first before being serially diluted (0–20 µg/mL in blocking buffer), and 100 µL of each dilution was incubated with the immobilized Hyperphage for 2 h. The depleted IgG samples were transferred to the ELISA plate with the immobilized SeM ORF phages and incubated for 2 h. Binding of IgGs to SeM ORFs was detected by goat anti-horse-HRP antibody (Novus Biologicals, 1:10,000 dilution) after incubation for 1 h, followed by incubating with TMB (Sigma) for 10 min. Binding is expressed as the absorbance (OD 450 nm) obtained for the binding to SeM ORFs minus absorbance for binding to the Hyperphage control (binding to the latter was typically <0.2 absorbance units). All horse sera IgGs were quantified using a horse IgG ELISA kit (AFG Bioscience). Commercial IgG samples used include horse IgG isotype control (10 mg/mL, NBP1-97045, Novus Biologicals), horse Fc native protein (1 mg/mL NBP1-97034, Novus Biologicals), and human IgG isotype control (5 mg/mL, 02-7102, Invitrogen).

## RESULTS AND DISCUSSION

### Panning horse serum with an *S. equi* ORFeome display library identified three major immunogenic surface proteins

An ORFeome display library was constructed for *S. equi* strain SEE_19-033, which possesses a 2.2 Mbp genome (GenBank accession number JAHRJJ000000000). The fragmented genomic DNA was cloned into the pHORF3 phagemid vector, and the open reading frames (ORFs) were displayed on the M13 phage upon packaging (37). The packaged ORFeome phage display library had an insert rate of 95%, and mapping of the library sequencing reads indicated that 100% of the SEE_19-033 genome was covered by ORF inserts (9,657,445 150 bp reads were aligned) (Data S6). The packaged phage display library was subsequently utilized for screening against 17 distinct equine serum samples, including commercial hyperimmune sera with strong serological responses toward *S. equi*, sera from horses that tested positive for *S. equi* infection, and sera from horses with known exposure to strangles (Table 1; Data S3). After two rounds of panning, ORF clones demonstrating binding to sera were cultured as monoclonal phage preparations and subjected to screening ELISA against sera to confirm their binding activity. ORF sequences of the clones with confirmed serum binding activity were determined, and the SEE_19-033 proteins corresponding to these ORFs were identified. A summary of the panning results from the 17 equine serum samples is shown in Table 1. Wide inter-animal variability in reaction to the ORFeome library was observed, with some samples (e.g., horses 14 and 17) showing high numbers of positive ELISA hits, and two horse samples (horses 6 and 9) not yielding any clones that showed immunogenic reactions in the screening ELISA. This highlights the variation in B-cell-derived immune answer in the horses tested due to the differences in *S. equi* infection/exposure status, as well as the innate biological differences among individual horses. By aligning all the immunogenic ORF sequences to the SEE_19-033 genome sequence, three major proteins were identified, which correlate with at least 10 ORFs identified from at least four different serum samples (Table 2). All three major immunogenic proteins are LPXTG-anchored surface proteins (38): a novel protein with proline-rich repeat domains, a serine protease, and the well-characterized surface protein SeM (18). Additionally, three minor proteins, characterized by fewer than 10 ORF hits and/or from fewer than four equine samples, were also identified (Table 2).

**TABLE 1:** Summary of panning results against 17 equine samples

Sample ID^*a*^	Sample type	No. of total ELISA positives	No. of different protein hits to the parental genome
1	Exposed to strangles^*b*^	7	6
2	Exposed to strangles^*b*^	13	7
3	Exposed to strangles^*b*^	12	2
4	Clinical case	6	1
5	Clinical case	19	5
6	Exposed to strangles^*b*^	0	0
7	Clinical case	2	1
8	Clinical case	27	7
9	Exposed to strangles^*b*^	0	0
10	Exposed to strangles^*b*^	8	7
11	Clinical case	6	6
12	Clinical case	18	6
13	Clinical case	2	1
14	Clinical case	13	8
15	Commercial^*c*^	17	7
16	Commercial^*c*^	5	5
17	Clinical case	48	8

^*a*^ See Data S3 for sample details.

^*b*^ Collected from horse with known exposure to strangles and received autogenous vaccine.

^*c*^ Commercial hyperimmune serum samples with strong serological responses to *S. equi*.

**TABLE 2:** *S. equi* proteins identified from ORFeome library panning and screening ELISA

Protein category	Protein NCBI accession	Protein (size)	Total number of ORFs alligned	Total horse samples detected
Major proteins (N-terminal signal peptide and C-terminal LPXTG-anchored cell surface proteins)	MCD3496749.1	Proline-rich repeat domain surface protein (417 aa)	82	9
	MCD3496188.1	S8 family serine peptidase (1634 aa)	10	6
	MCD3495510.1	SeM (534 aa)	10	4
Minor proteins	MCD3495726.1	CHAP domain-containing protein (392 aa)	3	3
	MCD3497125.1	Thioester-forming surface-anchored protein (546 aa)	5	4
	MCD3495701.1	IMP dehydrogenase (493 aa)	11	2

### A novel proline-rich repeat domain surface protein is highly conserved in *S. equi* but not in *S. zooepidemicus*

**Fig 1:**
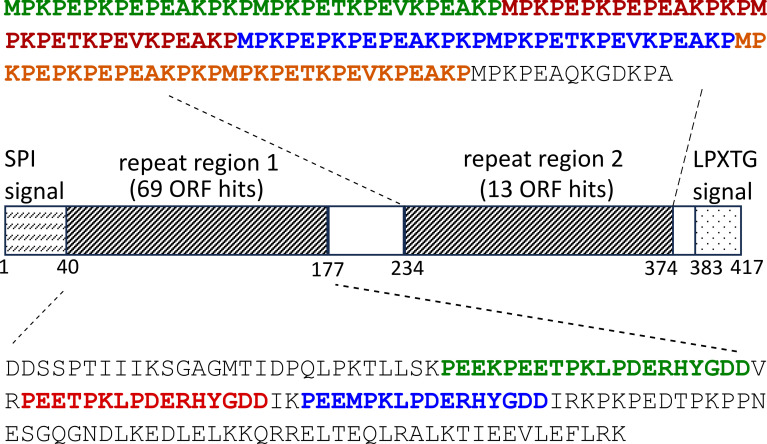
Mapping of immunogenic ORFs to the proline-rich repeat domain surface protein (accession number MCD3496749.1). A total of 82 ORFs showing binding to nine different horse sera used in panning were sequenced, and their sequences were aligned to the protein. The sequence repeat motifs in the aligned regions are highlighted.

Among the three major immunogenic proteins identified by ORFeome display, the protein with the highest number of aligned ORFs is a 417-amino acid (aa) protein containing an LPXTG cell wall anchor domain (accession number MCD3496749.1), which matched a total of 82 ORFs recovered from panning nine different horse samples (Table 2). This 417-aa protein is predicted to have a mature length of 347 aa (39.4 kDa) following processing at its N-terminal SPI and C-terminal LPXTG sortase signals (Fig. 1). This protein is characterized by the presence of two repetitive sequence regions with all 82 ORFeome sequences recovered from this protein located in one of these two regions (Fig. 1). The first immunogenic region spans position 40–177 and contains three copies of the imperfect repeat PEEXPKLPDERHYGDD, and the second immunogenic region spans positions 234–374 and comprises a proline-rich domain with four tandem repeats of the sequence KPEPKPEPEAKPKPMPKPETKPEVKPEAKN. This protein is predicted to be largely disordered based on analysis in JPred4 (39) and Alphafold 3 (40), with a small central coiled-coil domain spanning residues 150–178, the C-terminal portion of the first immunogenic sequence repeat domain. It is noteworthy that in addition to this protein (accession MCD3496749.1, or WP_012679405.1, 417-aa), the *S. equi* SEE 19_033 genome encodes another surface protein nearly identical (99% identity) to the previously characterized *S. equi* SzPSe protein (accession AAB71985.1, 374-aa) (16). Outside of the short, highly charged proline-rich (PEPK) repeats in the C-terminal region, SzPSe does not share sequence homology with the protein MCD3496749.1 and is lacking both immunogenic regions and the larger repeat motifs identified in this study. Previous work has also shown that these charged proline-rich (PEPK) repetition peptides from SzPSe react more strongly with equine sera taken from *S. equi*-challenged horses than horses infected with *S. zooepidemicus* (25, 41), suggesting these charged, disordered domains may play a role in the pathogenesis of or immune response to *S. equi*.

**Fig 2:**
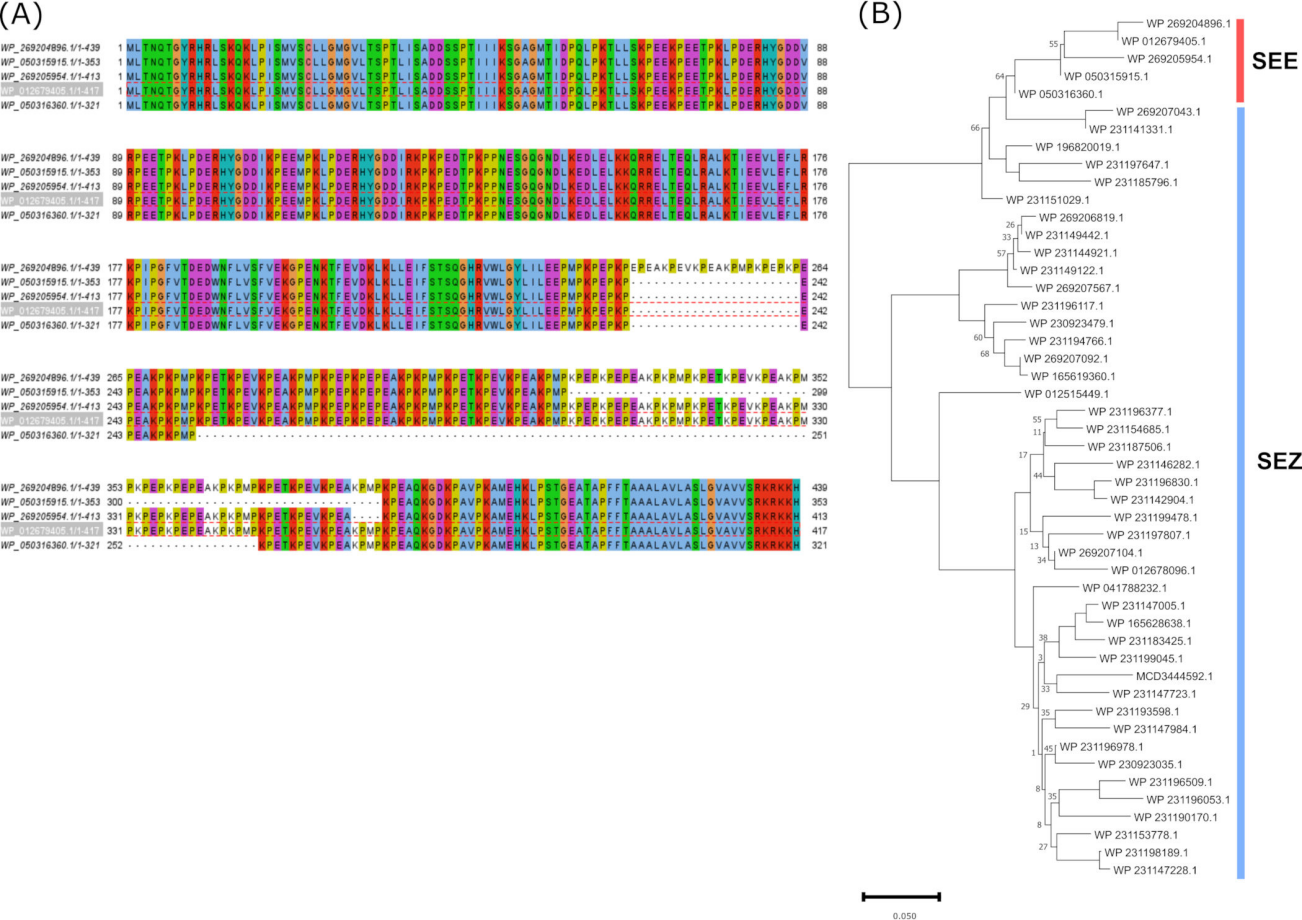
The proline-rich repeat domain surface protein is highly conserved in *S. equi,* but not in *S. zooepidemicus*. (**A**) The alignment of homologs identified from all *S. equi* genomes, which include 50 Texas isolates and 12 global isolates. (**B**) The phylogenetic tree of all homologs identified in all *S. equi* (SEE) and *S. zooepidemicus* (SEZ) genomes used in this study. The gene start for WP_269204896.1 and WP_269205954.1 was adjusted to include the signal peptide sequence for sequence alignment purposes. Only bootstrap values less than 70 are labeled next to the nodes.

A diverse collection of *S. equi* genomes was assembled, including 50 strains isolated from various regions of Texas, USA (NCBI Bioproject PRJNA736470) (38), and 12 strains that represent six genetically distinct Bayesian analysis of population structure (BAPS) groups and six multilocus STs based on a previous pairwise core-genome multilocus sequence typing (cgMLST) analysis of 670 global *S. equi* isolates (42). Detailed *S. equi* genome information is listed in Data S1. Protein alignment of the two identified immunogenic regions against these diverse *S. equi* genomes showed that these regions are highly conserved in *S. equi*. After standardizing the gene starts to include the N-terminal signal peptide sequences, all homologs of this protein in *S. equi* genomes could be condensed to one of five versions of this protein in the nonredundant NCBI reference sequence database (see Data S1 for accession numbers). These five alleles differ only in the second repeat region at the C-terminal end in the number of copies of the repeat (see Fig. 2A for sequence alignment). With the first immunogenic region 100% identical in all *S. equi* genomes included in this study, this analysis indicates that this surface protein is highly conserved among global strangles-associated *S. equi* strains.

A similar analysis was conducted for homologs of this protein in *S. zooepidemicus*, using 50 diverse *S. zooepidemicus* strains isolated from Texas, USA (from Bioproject PRJNA736470), representative of the differing disease states recognized for *S. zooepidemicus* in horses (i.e., commensal and virulent isolates) (43). In addition, three *S*. *zooepidemicus* isolates were retrieved from the NCBI that have complete genome assemblies, and strains ATCC35246 (44), MGCS10565 (45), and H70 (46) were also included (Data S2). In contrast to the homogeneity of this protein in *S. equi* genomes (Fig. 2A), high levels of variation were seen in both sequence-repeat regions among the *S. zooepidemicus* homologs, with each homolog sharing <86% overall sequence homology to the reference protein from *S. equi* strain 19-033 (accession MCD3496749.1 or WP_012679405.1). The diversity of these protein homologs in *S. zooepidemicus* and *S. equi* is illustrated in Fig. 2B. More *S. zooepidemicus* isolates representative of the global population diversity based on the phylogenetic analysis of isolates from Iceland and other regions (47) were also examined for the presence of this protein. However, only partial sequences of this protein (residues 1–254) could be identified in most *S. zooepidemicus* genome records because genome assemblies were interrupted, possibly due to the repetitive nature of the sequence after residue 254. Consistent with the alignment results using the available full versions of the proteins shown in Fig. 2B, the partial protein sequences also revealed great alignment variation in sequences from residues 1–254 (data not shown). *S. equi* and *S. zooepidemicus* share 98% DNA homology and also have similar virulence factors and morphological traits, making differential diagnosis challenging (48). Since *S. zooepidemicus* can be isolated from the upper respiratory tract of healthy horses (49), tests that do not distinguish between *S. equi* and *S. zooepidemicus* can lead to false positives for strangles. The high conservation of this surface protein sequence in global *S. equi* isolates, but not in *S. zooepidemicus*, makes this protein an attractive candidate as a diagnostic biomarker for differentiating *S. equi* from *S. zooepidemicus* as a complement to the current typing methods.

### The proline-rich repeat domain surface protein significantly reacts with *S. equi,* but not *S. zooepidemicus* sera

To validate the immunogenicity of the proline-rich repeat domain protein, the purified protein was validated for its ability to interact with equine sera collected from multiple horses. The sera tested include 25 samples from horses with diagnosed strangles, which were confirmed to be positive for *S. equi* by microbiologic culture, PCR, or both (designated as *S. equi* (+) in this study). As a comparison, 12 control sera from healthy horses without exposure to *S. equi* and with no history of strangles or exposure to infected horses were also evaluated. Furthermore, 15 sera from horses that tested negative for *S. equi* by microbiologic culture and PCR, but positive for *S. zooepidemicus* by microbiologic culture or PCR (designated as *S. zooepidemicus* (+) in this study)*,* were also tested (Data S3). All horse sera had similar total IgG levels (ranging from 1.75 to 2.50 mg/mL), as determined using a horse IgG quantification kit. Figure 4A shows the reactivity of this recombinant protein to *S. equi* (+) versus the control sera, represented by EC50 values, which were calculated as the serum dilutions that resulted in the half-maximum of the ELISA signals and is a reflection of the titer of antibodies specific to this protein. A significantly (*P* < 0.0001) stronger reaction (5-fold lower mean EC50 value) to this protein was observed for the *S. equi* (+) sera relative to the control sera, indicating this protein is immunogenic in horses with strangles. In contrast, the reactivity to this protein in serum samples from horses infected with *S. zooepidemicus* was not significantly different (2-fold lower mean EC50 value, *P* = 0.0566) than that of control horses. Using an EC50 cutoff value of 0.000138 (1:7246 dilution of the tested sera, or 242–345 ng IgG/mL), the specificity of this recombinant proline-rich protein in detecting strangle (SEE+) is 100% (95% CI = 77.9%−100%), and the sensitivity is 88% (95% CI = 67.9%−97.4%) (Data S4). It is worth noting that the detection tests reported here utilized the full-length recombinant protein and do not represent a final clinical assessment of the diagnostic sensitivity and specificity. The high level of sequence conservation of this surface protein in global *S. equi* isolates, coupled with its distinct immunogenicity in horses with strangles as reported here, suggests the use of this protein as a diagnostic marker for *S. equi*, and also potentially as a vaccine candidate for equine strangles. A more rigorous evaluation using only the peptide sequences in the immunogenic regions of this protein and testing against a larger collection of sera is needed for future study.

### The cell surface protease is highly conserved in *S. equi* but diverged as two groups in *S. zooepidemicus*

**Fig 3:**
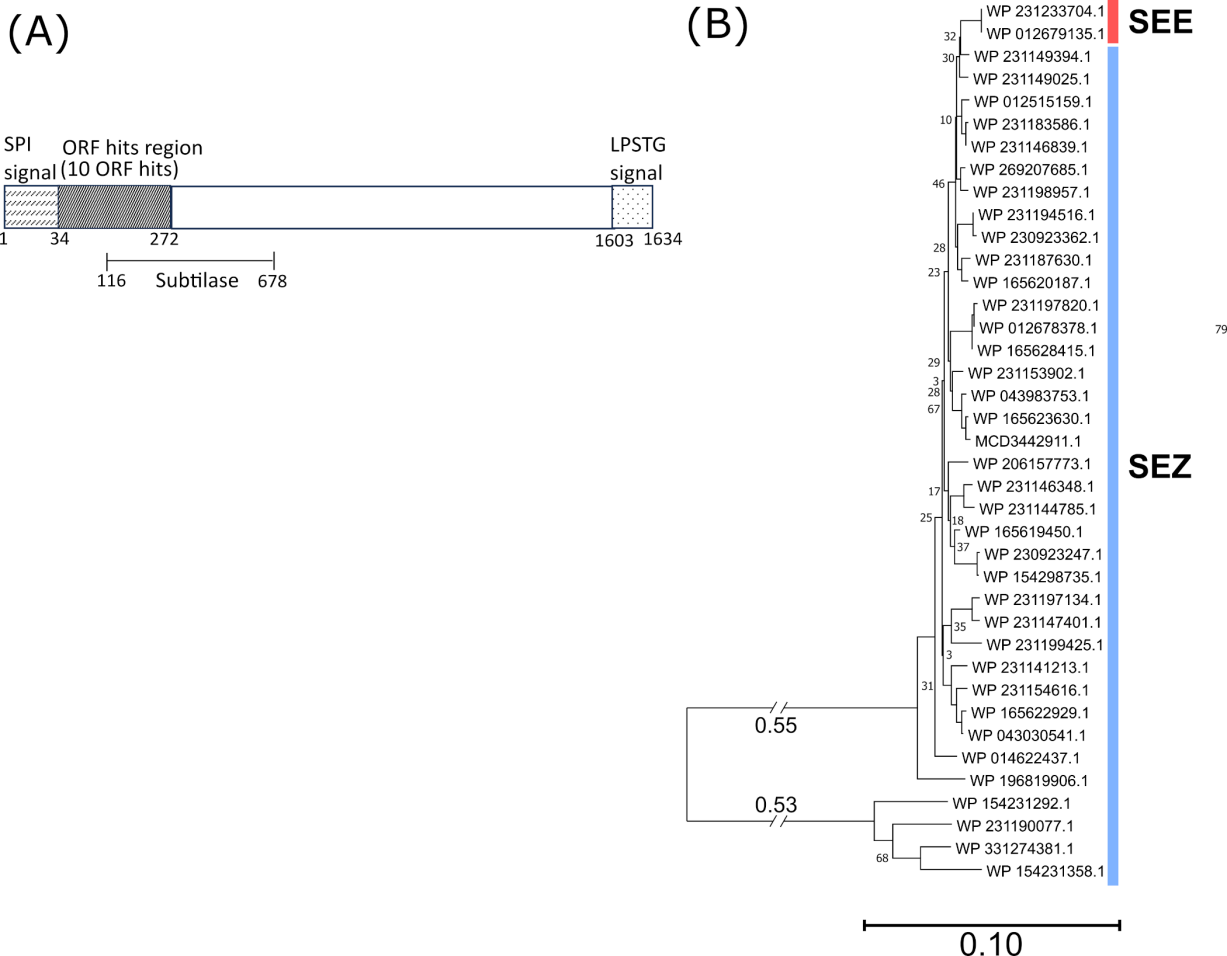
Mapping of the immunogenic ORFs to the serine peptidase and the homologs in *S. equi* and *S. zooepidemicus*. (**A**) A total of 10 ORFs showing binding to six different horse sera used in panning were sequenced, and their sequences were aligned to the protein. Residue positions of key domains are labeled. (**B**) The phylogenetic tree of all homologs identified in all *S. equi* (SEE) and *S. zooepidemicus* (SEZ) genomes used in this study. Only bootstrap values less than 70 are labeled next to the nodes.

The second major surface protein identified from ORFeome Phage Display panning is a 1634-aa S8 family serine peptidase (accession number MCD3496188.1 in *S. equi* strain 19-033; included in WP_12679135), which matched a total of 10 ORFs identified from panning against six different *S. equi* (+) serum samples (Table 2). These identified ORFs mapped onto the N-terminal sequence region from residues 42 to 272, directly downstream of the signal peptide sequence (residues 1−34), and partially covering the subtilisin-serine protease domain region (116−678, InterPro IPR015500) (Fig. 3). This protein also contains a C-terminal LPSTG cell wall-anchoring motif for secretion and anchoring of this enzyme to the cell surface. The BLASTp analysis of this protein against all *S. equi* genomes included in this study (Data S1) indicates the identical homologs (clustered as one protein group WP_012679135.1) in all *S. equi* genomes, except one (strain 18-008, protein accession WP_231233704.1), which only has two residue insertions (at position 1,123) to the consensus homolog (WP_012679135.1). This protein is also highly conserved (95%−99% homology to the *S. equi* consensus sequence WP_012679135.1), in 46 out of 53 total *S. zooepidemicus* genomes examined in this study (Data S2). In the rest of the *S. zooepidemicus* genomes (7 out of 53), the surface-anchored peptidase harboring the same N-terminal subtilisin-serine protease domain (InterPro IPR015500) appears as a truncated version (1,115 aa−1,118 aa in length, Data S2). The diversity of this protein in *S. zooepidemicus* genomes and the contrast to the sequence conservation in *S. equi* genomes are illustrated in Fig. 3B. The longer protease version (~1,634 aa) and the shorter version (~1,118 aa) form two distinct groups, with high sequence homology within each group but low sequence homology across the protein length, including the immunogenic N-terminal region, between these two groups (Fig. 3B; Data S5). The N-terminal immunogenic region of this protein (residues 42−272) only shared 30%−35% identity to the aligned regions (residues ~ 79 to 188) in the truncated versions. These data suggest that using the sequence alone, this surface protease may not adequately differentiate *S. equi* from *S. zooepidemicus* in diagnostic typing applications.

### The cell surface protease is a homolog of the *S. pyogenes* SpyCEP and shows differential reactivity toward *S. equi* vs *S. zooepidemicus* sera

This cell surface protease protein, highly conserved in all *S. equi* strains tested, shared 62% sequence homology over the whole length of the protein (100% probability via HHpred alignment) with the highly conserved cell surface serine protease SpyCEP (PDB code 6VJB, NCBI accession ABD72239.1, or identical protein group WP_023613070.1) from the human pathogen *S. pyogenes*. SpyCEP has been shown to inactivate chemokines and impair neutrophil recruitment and bacterial clearance (50, 51). The equine IL-8 cleavage function has been reported with the cell surface serine protease of *S. equi* strain 4047 (conserved in *S. equi* as WP_012679135.1) and *S. zooepidemicus* strain H70 (98% sequence identity to *S. equi* consensus homolog WP_012679135.1). The *S. equi* surface protease has been proposed as a vaccine candidate as it has shown promising immunogenicity and protective activity in mouse challenge models of Group A *Streptococcus* infection (52–54). Similarly, the partial SpyCEP protein from *S. pyogenes* (comprising amino acid residues 35−587, Genbank No. DQ413032) when expressed exhibited cross-protectivity against infection dissemination of *S. equi* (55).

**Fig 4:**
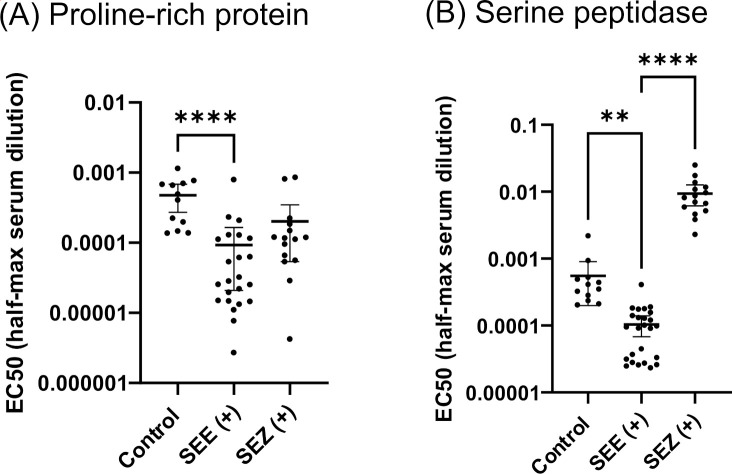
Reactivity of the (**A**) proline-rich repeat domain surface protein, and the (**B**) serine peptidase, with equine sera. EC50 values (the serum dilutions resulting in half-maximum ELISA signals) of the recombinant protein reacting with each serum sample are determined by ELISA and plotted. The mean of the EC50 values and 95% CI of mean within each serum group are shown, and pair-wise statistical significance is indicated. **P = 0.0025; ****P < 0.0001. Control: sera without known infection and exposure to *S. equi*; SEE(+) and SEZ (+): sera from horses tested positive for *S. equi* (SEE) or *S. zooepidemicus* (SEZ).

The complete sequence of this *S. equi* surface protease was expressed in *E. coli,* and the purified protein was tested for its reactivity with equine sera (Fig. 4B). The reaction of this protein to the *S. equi* (+) sera was significantly stronger (*P* = 0.0025), as indicated by a 5-fold lower mean EC50 value (serum dilution resulting in half-maximum ELISA signal) relative to the control sera. This protein reacted significantly (*P* < 0.0001) more weakly (evident by a 90-fold higher mean EC50 value) to *S. zooepidemicus* (+) sera compared to the *S. equi* (+) sera, indicating the ability of this protein to differentiate between infections caused by these two bacteria. Using an EC50 cutoff value of 0.000203 (1:4,926 dilution of the tested sera, or 355−506 ng IgG/mL), the specificity of this surface protease in detecting strangle (SEE+) is 100% (95% CI = 77.9%−100.0%), and the sensitivity is 96% (95% CI = 79.6%−99.9%) (Data S4). The observed differential interaction of this surface protease with *S. equi* vs *S. zooepidemicus* sera, coupled with the immunogenicity of this protease reported in the literature, highlights the potential value of this protein as a diagnostic marker with specificity for strangles or as a strangles vaccine candidate. The ORF hit region identified in this study covers a relatively short N-terminal sequence region (residues 42−272), suggesting the presence of a key immunogenic epitope within this region. Future work is needed to test only the immunogenic region of this protease against a larger sample size of sera to make a final clinical assessment of this protein.

### Validation of the immunogenicity of SeM ORFs

**Fig 5:**
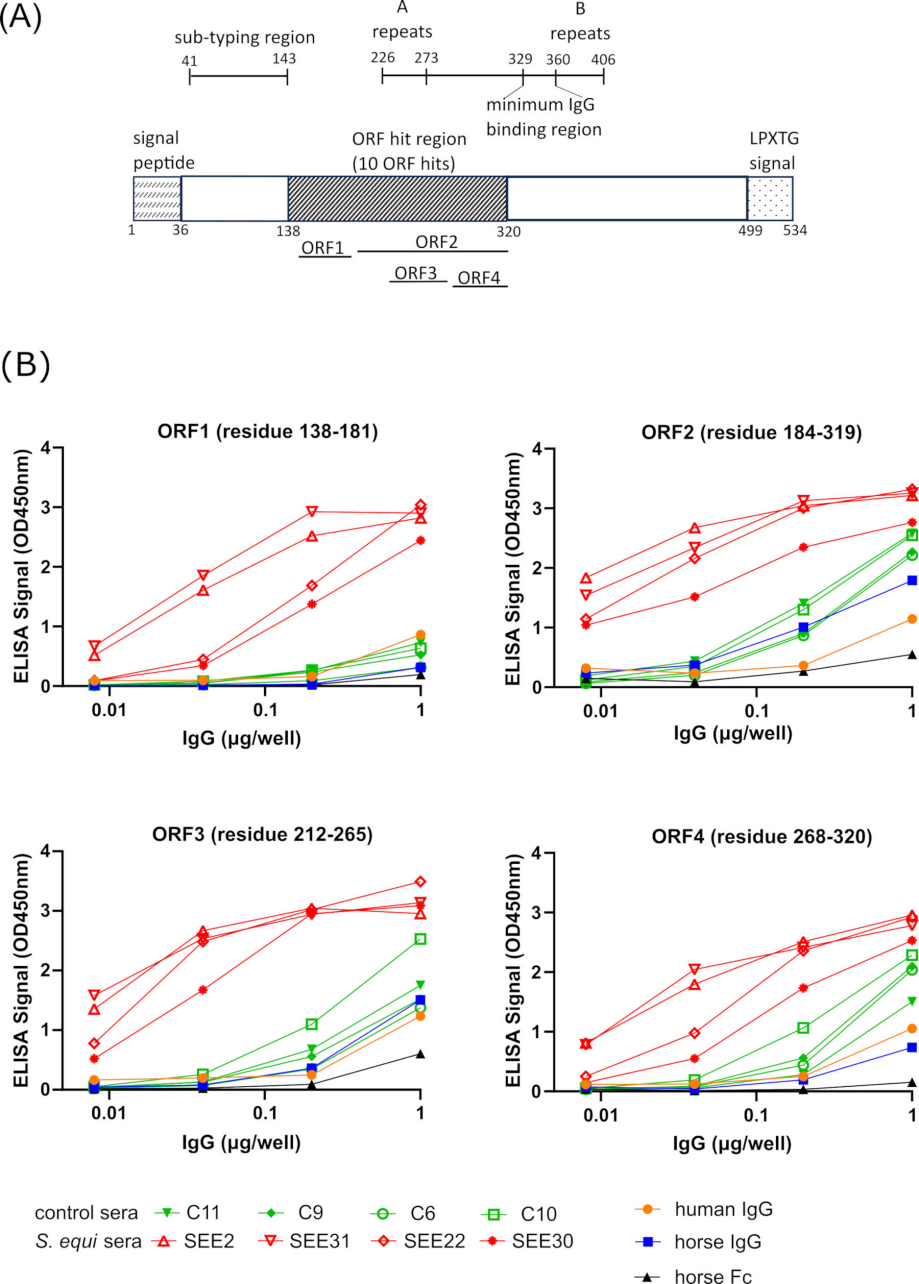
Mapping of the immunogenic ORFs on the SeM protein. (**A**) A total of 10 ORFs showing binding to four different horse sera used in panning were sequenced, and their sequences were aligned to the protein. Residue positions of key domains are labeled. (**B**) Interaction of four ORFs with different IgG samples was determined by ELISA with standardized IgG inputs. Samples tested include horse and human IgG isotype controls, horse IgG-Fc, four sera from control horses without known infection and exposure to *S. equi* (C6, 9, 10, and 11), and four sera from horses tested positive for *S. equi* (SEE2, 22, 30, and 31), see Data S3 for more information.

Another major protein that was identified in this study was a 534-aa protein (accession number MCD3495510.1 in the *S. equi* 19-033 genome, identical protein WP_231192941.1), which is the well-characterized *S. equi* virulence determinant and immunogenic protein SeM (16, 56), also termed fibrinogen-binding protein (FgBP) (57). A total of 10 ORFs identified from panning against four different serum samples were mapped onto this protein (Table 2). It is known that SeM binds to fibrinogen and immunoglobulin G (IgG) (14, 16, 18, 57, 58) and exhibits an anti-phagocytic effect comparable to that of the M proteins of group A streptococci (12, 13). The protein SeM binds strongly to only two of the seven eqIgG subclasses (eqIgG4 and eqIgG7), and like other streptococcal and staphylococcal IgG- and IgA-specific Ig-binding proteins, SeM binds to the domain interface of the IgG-Fc region (57, 59). Due to the native binding ability of SeM to IgG, the SeM ORFs identified in this study were validated with caution. Similar to the other cell surface proteins identified in this study, SeM possesses an N-terminal SPI signal and a C-terminal LPSTG sorting signal (Fig. 5A). Using recombinant SeM proteins with defined internal deletions, it has been reported that the minimal SeM IgG-binding site (determined to be residues 329−360) is centrally located between the A and B repeat sequences, with residues 335−348 critical for binding (57). In this study, the individual SeM ORF clones identified through panning and screening span residues 138−320, which is outside of the reported critical IgG-binding region (Fig. 5A). Even though the *S. equi* ORFeome library is depleted for the nonspecific binders including those to IgG-Fc, the ORFs identified here were further validated in order to determine if the interaction of these ORFs to horse sera was due to their immunogenicity rather than non-immune IgG interaction intrinsic to the SeM protein. Four representative SeM ORFs spanning the identified region were tested in ELISA assays against different IgG samples including purified horse IgG and human IgG isotypes, horse IgG-Fc, as well as horse sera from control and *S. equi* groups (four samples each, Fig. 5B). ELISA assays were conducted using mouse and goat antibodies (as anchoring and detection antibody, respectively), which are known to not react with SeM (57). The input IgGs or Fc quantity was standardized, and their binding to the SeM ORFs was determined at different dilutions. All four tested ORFs showed a stronger reaction to *S. equi* (+) sera than with control sera. In addition, these ORFs showed minimum binding to human IgG, horse IgG, and horse Fc, distinct from the reported strong binding of full-length SeM to the Fc fragment of horse and human origin (57). Together, these results indicate binding of serum antibodies to an immunogenic region of the *S. equi* SeM protein rather than nonspecific IgG binding by SeM fragments.

Following proper library and sera pre-incubation steps to deplete nonspecific binders, the identification of SeM by the ORFeome phage display approach in this study is consistent with previously established immunogenic roles of SeM and thus validates this technology pipeline for the discovery of immunogenic proteins. Because SeM binds strongly to Fc fragments of equine origin (26, 57), which could lead to false-positive results in ELISA, validation of the full-length SeM for its binding toward sera was not conducted using ELISA in this study. The region identified by ORFeome mapping lies upstream of the critical IgG-binding domain of SeM and is consistent with previous reports that epitopes reactive with convalescent equine IgG and mucosal IgA were concentrated toward the conserved center region of SeM (19, 57). Future work is needed to investigate the immunogenic epitopes in the identified ORF sequences here, which may be useful to complement existing immunogenic strangles assays including the antigen A and antigen C ELISA assay (26).

In summary, the utilization of the ORFeome display platform as a high-throughput screening method for immunogenic peptides of *S. equi* that interact with sera from horses with strangles has led to the identification of three major immunogenic proteins. Two of these proteins (the serine protease and SeM) have been previously reported to possess immunogenic activity, and the third protein identified in this study appears to be a novel protein carrying two proline-rich sequence repeat domains and shares no homology to previously described SzPSe protein. This study further identified the immunogenic region in the serine peptidase as the N-terminal 272 residues and the immunogenic region of SeM as residues 138−320, adjacent to the protein’s critical IgG-binding domain. Notably, these proteins are all bacterial surface proteins carrying a signal sequence at the N-terminus and a gram-positive anchor domain with the LPXTG sortase processing site at the C-terminus. This finding is consistent with the well-established role of surface proteins in most gram-positive bacteria in facilitating interactions between cells and their environment. These surface proteins often function as virulence determinants, such as agglutinins and adhesins, impeding phagocytosis and preventing opsonization (38). Both the proline-rich repeat domain protein and the serine protease showed high levels of sequence conservation in global *S. equi* isolates but not in *S. zooepidemicus*, supporting previous evidence that *S. equi* is a clonal pathogen originating from an ancestral strain of *S. zooepidemicus*, and isolates of *S. equi* are antigenically and genetically similar (15, 60). The high degree of sequence conservation in *S. equi*, and not in *S. zooepidemicus*, in conjunction with the robust differential interaction with the strangle positive sera, suggests the potential for the proline-rich repeat domain protein and the serine protease protein to serve as diagnostic markers or vaccine candidates for equine strangles caused by *S. equi*. Further clinical assessment using only the immunogenic regions of these proteins against a larger sample size of sera is needed to achieve optimal and robust diagnostic sensitivity and specificity.

## SUPPLEMENTAL MATERIAL

Data S1 msphere.00626-25-s0001.xlsx Collection of *S. equi* strains used in this study.

Data S2 msphere.00626-25-s0002.xlsx Collection of *S. zooepidemicus* strains used in this study.

Data S3 msphere.00626-25-s0003.xlsx List of all serum samples used in this study.

Data S4 msphere.00626-25-s0004.xlsx Sensitivity and specificity of the proline-rich protein and S8 protease reacting with sera from *S. equi* infections.

Data S5 msphere.00626-25-s0005.png Sequence alignment of all serine protease homologs in *S. equi* and *S. zooepidemicus*.

Data S6 msphere.00626-25-s0006.pdf Mapped coverage depth of ORFeome insert library against the *S. equi* strain SEE19-033 genome.

Data S7 msphere.00626-25-s0007.txt FASTA sequences of all proline-rich protein homologs in SEE and SEZ.

Data S8 msphere.00626-25-s0008.txt FASTA sequences of all serine peptidase homologs in SEE and SEZ.

ASM does not own the copyrights to Supplemental Material that may be linked to, or accessed through, an article. The authors have granted ASM a non-exclusive, world-wide license to publish the Supplemental Material files. Please contact the corresponding author directly for reuse.
