# Supplementary figures and images for: Immunogenic Streptococcus equi cell surface proteins identified by ORFeome phage display
Source: mSphere. 2025 Nov 25;10(12):e00626-25. doi: 10.1128/msphere.00626-25 (PMC12724188; doi:10.1128/msphere.00626-25)

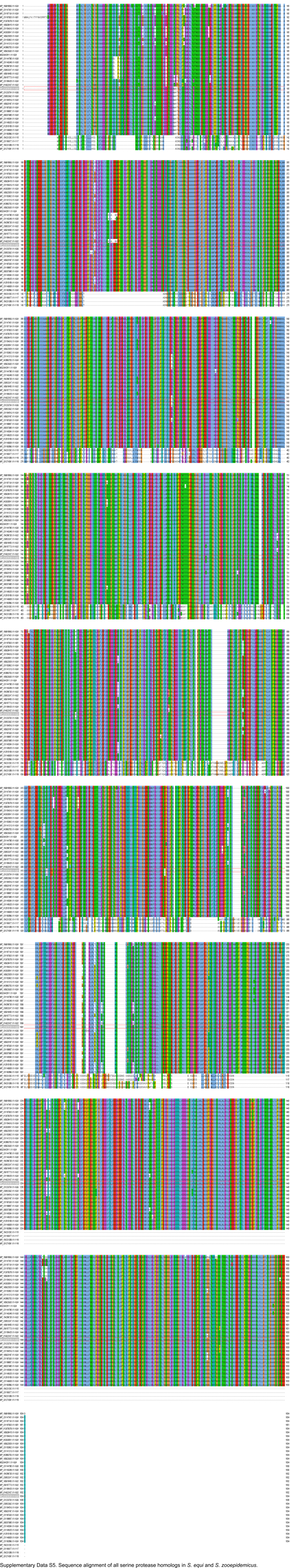

Supplement: Data S5 — Sequence alignment of all serine protease homologs in S. equi and S. zooepidemicus. [file msphere.00626-25-s0005.png]
