# Supplementary material for: Immunogenic Streptococcus equi cell surface proteins identified by ORFeome phage display
Source: mSphere. 2025 Nov 25;10(12):e00626-25. doi: 10.1128/msphere.00626-25 (PMC12724188; doi:10.1128/msphere.00626-25)
Supplement: Data S6 — Mapped coverage depth of ORFeome insert library against the S. equi strain SEE19-033 genome. [file msphere.00626-25-s0006.pdf]

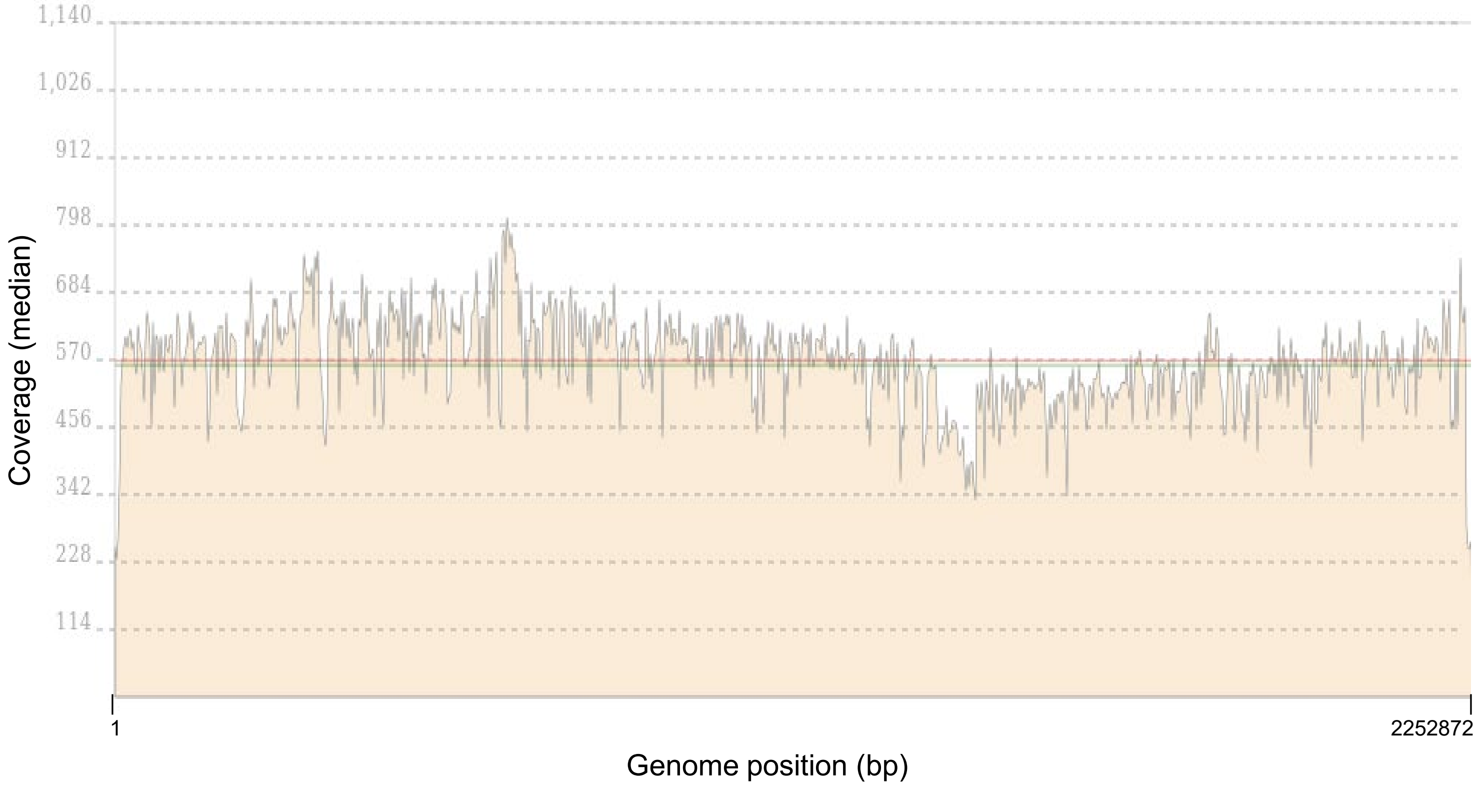

Supplementary Data S6. Mapped coverage depth of ORFeome insert library against the *S. equi* strain SEE19-033 genome. The constructed ORFeome libraries (pHORF3 phagemid vector carrying the ORFs inserts) were sequenced using NovaSeq Illumina sequencing to generate 2x150bp paired-end reads. The obtained reads (a mixture of the ORF inserts and the pHORF3 vector, total 236,585,138 reads) were mapped onto the *S. equi* strain SEE_19-033 genome using Bowtie2 (9,657,445 reads were aligned), and the resulting BAM file was used to plot the mapped coverage depth of the ORF insert reads against the SEE19-033 genome by WGSCoveragePlotter. There is a ~2.4-fold difference between the maximum (~800 read counts) and minimum (~340 read counts) coverage depth across the genome.
